# Convergent Plastome Evolution and Gene Loss in Holoparasitic Lennoaceae

**DOI:** 10.1093/gbe/evy190

**Published:** 2018-08-29

**Authors:** Adam C Schneider, Thomas Braukmann, Arjan Banerjee, Saša Stefanović

**Affiliations:** 1 Department of Biology, University of Toronto Mississauga, Ontario, Canada; 2 Department of Ecology and Evolutionary Biology, University of Toronto, Ontario, Canada; 3 Centre for Biodiversity Genomics, University of Guelph, Ontario, Canada; 4 Department of Biology, Hendrix College, Conway, AR

**Keywords:** Boraginales, gene loss, *Lennoa madreporoides*, parasitic plants, purifying selection, *Pholisma arenarium*, *Tiquilia plicata*

## Abstract

The Lennoaceae, a small monophyletic plant family of root parasites endemic to the Americas, are one of the last remaining independently evolved lineages of parasitic angiosperms lacking a published plastome. In this study, we present the assembled and annotated plastomes of two species spanning the crown node of Lennoaceae, *Lennoa madreporoides* and *Pholisma arenarium*, as well as their close autotrophic relative from the sister family Ehretiaceae, *Tiquilia plicata*. We find that the plastomes of *L. madreporoides* and *P. arenarium* are similar in size and gene content, and substantially reduced compared to *T. plicata*, consistent with trends seen in other holoparasitic lineages*.* In particular, most plastid genes involved in photosynthesis function have been lost, whereas housekeeping genes (ribosomal protein-coding genes, rRNAs, and tRNAs) are retained. One notable exception is the persistence of a *rbcL* open reading frame in *P. arenarium* but not *L. madreporoides* suggesting a nonphotosynthetic function for this gene. Of the retained coding genes, *d*_N_/*d*_S_ ratios indicate that some remain under purifying selection, whereas others show relaxed selection. Overall, this study supports the mounting evidence for convergent plastome evolution in flowering plants following the shift to heterotrophy.

## Introduction

Photosynthesis is the predominant carbon and energy acquisition strategy employed by autotrophs, but some plants (1–2% of angiosperms) survive by acquiring part or all of these necessary resources from other plants. This heterotrophy can occur indirectly, in which the parasitic plant associates with mycorrhizal fungi in order to exploit the fungal mutualism with autotrophic plants (mycoheterotrophy), or directly, in which the parasitic plant attaches to the vascular tissue of one or more host plants using specialized organs called haustoria. Among angiosperms, direct parasitism has evolved at least 13 times (Westwood et al. 2010; Su et al. 2015) and has, in most cases, led to the complete loss of autotrophy (i.e., holoparasitism). In nearly every parasitic plant lineage, the evolution of heterotrophy is associated with dramatic changes in morphology, life history, and genomic architecture, collectively referred to as the “parasitic reduction syndrome” (Colwell 1994). The independent origins of parasitism across the plant phylogeny provide an excellent opportunity to assess the level of convergence in various ecological, morphological, and genetic traits, as well as develop and test models that can predictably describe evolutionary trajectories of parasitic plants.

Particular attention has been given to changes in the chloroplast genome (plastome). As the chloroplast is the site of photosynthetic activity in plant cells, the plastomes of most plants unsurprisingly contain genes that code for key portions of the photosynthetic apparatus, along with housekeeping genes and several others with unknown function (Wicke et al. 2011; Braukmann et al. 2017). Generally, plastomes of heterotrophic plants have accumulated many more mutations and structural changes, and show substantial reductions in both sequence length and gene content compared with their highly conserved counterparts in closely related autotrophs. This is thought to be because of the relaxation of purifying selection on photosynthesis-related genes following the evolution of heterotrophy (Bellot and Renner 2015; Naumann et al. 2016; Wicke et al. 2016; Graham et al. 2017).

The earliest studies of plastome evolution in parasitic plants were in the Orobanchaceae (*Epifagus virginiana*, dePamphilis and Palmer 1990; Wolfe et al. 1992), and this clade continues to be one of several model systems for descriptive studies and broad syntheses leading to development of evolutionary theory (Wicke et al. 2013, 2016). At the same time, studies among many independently evolved holoparasite lineages are important to test the generalizability of patterns or processes identified in a particular system. This effort, to generate sequenced plastomes representing each origin of parasitism, is nearly complete, with published studies of species in the following clades: *Cassytha* (Lauraceae, Wu et al. 2017), Hydnoraceae (Naumann et al. 2016), Cynomoriaceae (Bellot et al. 2016), Apodanthaceae (Bellot and Renner 2015), Cytinaceae (Roquet et al. 2016), *Cuscuta* (Convolvulaceae, Funk et al. 2007; McNeal et al. 2007), Orobanchaceae (dePamphilis and Palmer 1990; Wolfe et al. 1992; Li et al. 2013; Samigullin et al. 2016; Wicke et al. 2016; Cho et al. 2018; Schneider et al. 2018), and the Santalales (Petersen et al. 2015).

Additionally, substantial sequencing effort of *Rafflesia lagascae* (Rafflesiaceae) by Molina et al. (2014) found fragments of several pseudogenized chloroplast genes and nongenic regions from the inverted repeats (IRs). However, no evidence of an intact plastid genome was found, from which the authors concluded that the plastome may be absent. The highly reduced plastome of *Mitrastemon* (Mitrastemonaceae) was described by Shyu (2013) in her Ph.D. dissertation; however, to the best of our knowledge, this research has not yet been formally published. The three remaining lineages comprise the holoparasitic Balanophoraceae, which is thought to have a highly reduced if not absent plastome (Nickrent et al. 1997), hemiparasitic *Krameria* (Krameriaceae), which appears to possess a near complete plastome (unpubl. data), and the holoparasitic Lennoaceae (Boraginales).

The Lennoaceae are a small, monophyletic family of herbaceous, achlorophyllous root parasites that grow from southwestern North America to northern South America (Yatskievych and Mason 1986; Boraginales Working Group 2016). From a morphological perspective, species in this clade show many of the same derived traits as other root holoparasites: vestigial, scale-like leaves, loss of a developed root system, and the reduction of the aboveground portion of the plant to a dense inflorescence. Species and populations of Lennoaceae generally have high levels of host specificity (Yatskievych 1982; Yatskievych and Mason 1986). However, potential convergence of molecular or genomic evolution is relatively unknown. In pursuit of the larger aim to identify shared evolutionary trajectories among parasitic plants, the primary objective of this study is to sequence, annotate, and compare the chloroplast genomes of two species that span the crown node of the Lennoaceae, *Pholisma arenarium* and *Lennoa madreporoides*, with a species from its autotrophic sister family, *Tiquilia plicata* (Ehretiaceae). Curiously, this species, along with its congener *T. palmeri*, are common hosts of their close parasitic relative *Pholisma sonorae* (Yatskievych and Mason 1986), a phenomenon referred to as adelphoparasitism*.* With these data, we seek to test the hypothesis, supported by evidence from other independently evolved lineages of parasitic plants, that plastome reduction is relatively advanced in holoparasites, including the complete loss of photosynthesis-related genes, and a relaxation of purifying selection on other genes.

## Materials and Methods

### DNA Extraction and Sequencing

Genomic DNA (gDNA) was extracted from ground floral tissue of single individuals of *Pholismaarenarium* Nutt. ex Hook and *Lennoamadreporoides* Lex., and leaf tissue of *Tiquiliaplicata* (Torr.) A.T.Richardson using a modified cetyltrimethylammonium bromide (CTAB) method (Doyle 1987). Voucher specimens were also made and deposited in registered herbaria (table 1). DNA extracted from *P. arenarium* was sent to Genome Quebec at McGill University in Montreal, Quebec for library preparation and high-throughput sequencing on their Illumina HiSeq 2000 platform using a 2x100 paired-end read format. DNA extracted from *L. madreporoides* and *T. plicata* was sent to The Centre for Applied Genomics at Sick Kids Hospital in Toronto, Ontario for library preparation and high-throughput sequencing on their Illumina HiSeq 2500 platform using 2x125 paired-end format. Raw reads for each sample were demultiplexed and the indexing barcodes removed by the sequencing facilities.

**Table 1:** Specimen and Voucher Data for Genomic Samples

Taxon	Collector & Collection Number	Herbarium^a^
*Tiquilia plicata*	Stefanović SS-16-23	TRTE
*Lennoa madreporioides*	Yatskievych et al. 83-370	IND131539
*Pholisma arenarium*	Alison Colwell s.n.	TRTE

a Index Herbariorum acronyms followed by accession number, if known.

### Plastome Assembly and Analysis

Quality trimming of raw reads was performed using Sickle v. 1.33 (Joshi and Fass 2011) with the threshold for quality set at a minimum PHRED score of 27 at each nucleotide and the threshold for minimum length at 71 bp per read for *P. arenarium* and at 99 bp per read for *T. plicata* and *L. madreporoides*.

The trimmed reads were assembled into contigs using the de novo assembly algorithm in Geneious v. 9.1.8 (Biomatters, Auckland, New Zealand; Kearse 2012). Several independent assemblies were performed using between 15 and 25 percent of the total trimmed read pool (21,695,598 reads for *L. madreporoides*, 24,674,638 for *T. plicata*, and 45,043,653 *for P. arenarium*)*.* Plastome contigs were then aligned and joined using the results of an independent NOVOplasty assembly (version 2.6, Dierckxsens et al. 2016). Aside from the low-quality ends, Geneious and NOVOplasty contigs had 100% sequence similarity. Finally, to confirm that contigs were joined correctly, the original read pool was reference-mapped against the de novo assembly using Geneious.

Plastome annotations of *Tiquilia* were performed in Geneious using several autotrophic angiosperms as references: *Arabidopsis thaliana*, *Nicotiana tabacum*, *Ipomoea nil*, and *Ipomoeatrifida* (Genbank accessions NC_000932, NC_001879, NC_031159, NC_034670). The annotated *Tiquilia* plastome was then added to the set of references above to annotate the rRNA and protein coding genes in *Pholisma* and *Lennoa*, with manual BlastX searches to confirm open reading frames (ORFs) (https://blast.ncbi.nlm.nih.gov/Blast.cgi). tRNA boundaries and anticodon identities were verified using tRNAscan v. 2.0 (Lowe and Chan 2016).

The ratio of nonsynonymous (*d*_N_) to synonymous (*d*_S_) substitutions of coding regions was calculated in *Lennoa* and *Pholisma* relative to *Tiquilia* to estimate the selection pressure acting on these genes. We used the Yang and Nielsen (2000) method implemented as the function yn00 in PAML v4.8 (Yang 2007). We classified genes as evolving under relaxed selection (*d*_N_/*d*_S_ > 0.7), weak purifying selection (0.3 < *d*_N_/*d*_S_ < 0.7), or purifying selection (*d*_N_/*d*_S_ < 0.3).

## Results

### Plastome Reduction in Lennoaceae

**Fig. 1.**
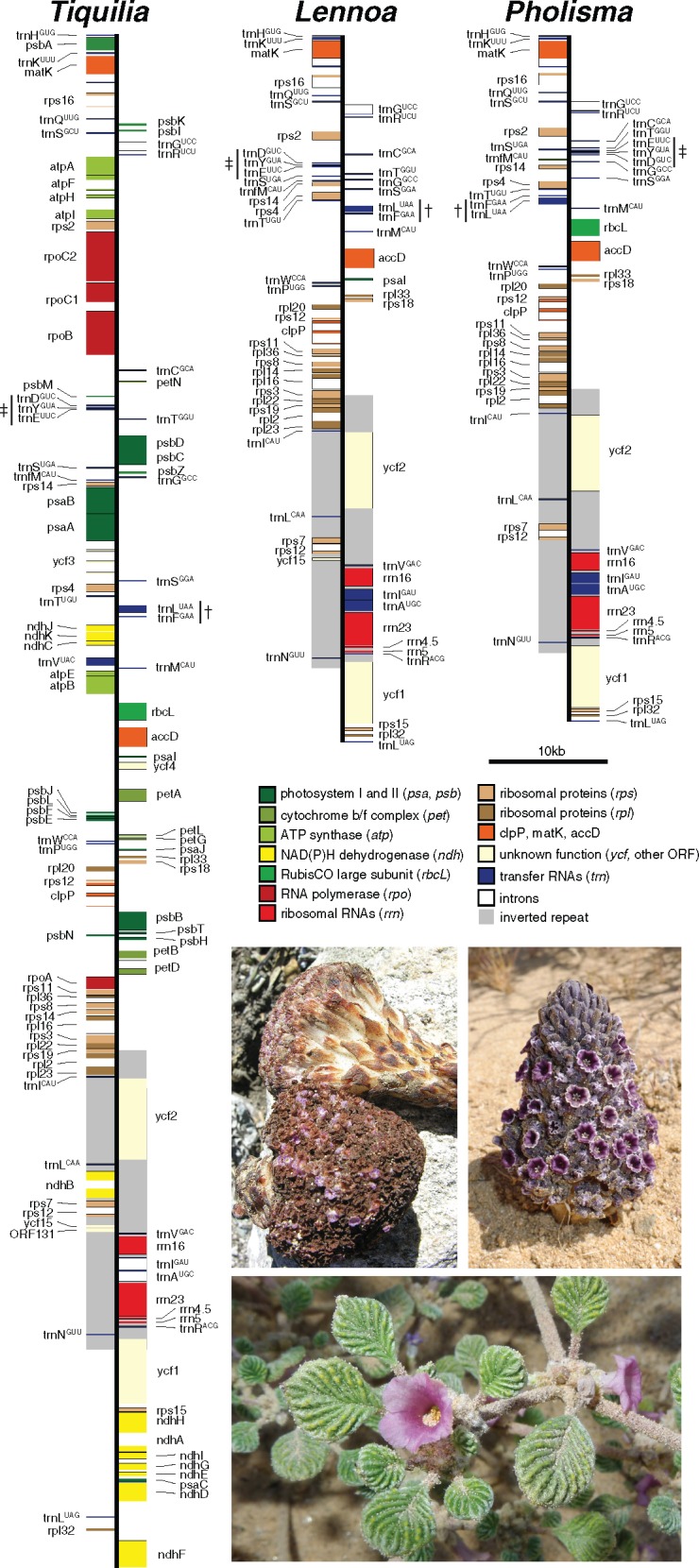
—Annotated chloroplast genomes of *Tiquilia plicata*, *Lennoa madreporoides*, and *Pholisma arenarium* (photos clockwise, from bottom). For concision, only one of the two inverted repeat regions are shown (gray background). Two structural rearrangements in the *P. arenarium* plastome relative to the other two species are indicated by † and ‡ respectively. Photos courtesy of Keir Morse (*T. plicata* and *P. arenarium*) and Dick Culbert (*L. madreporoides*).

**Fig. 2.**
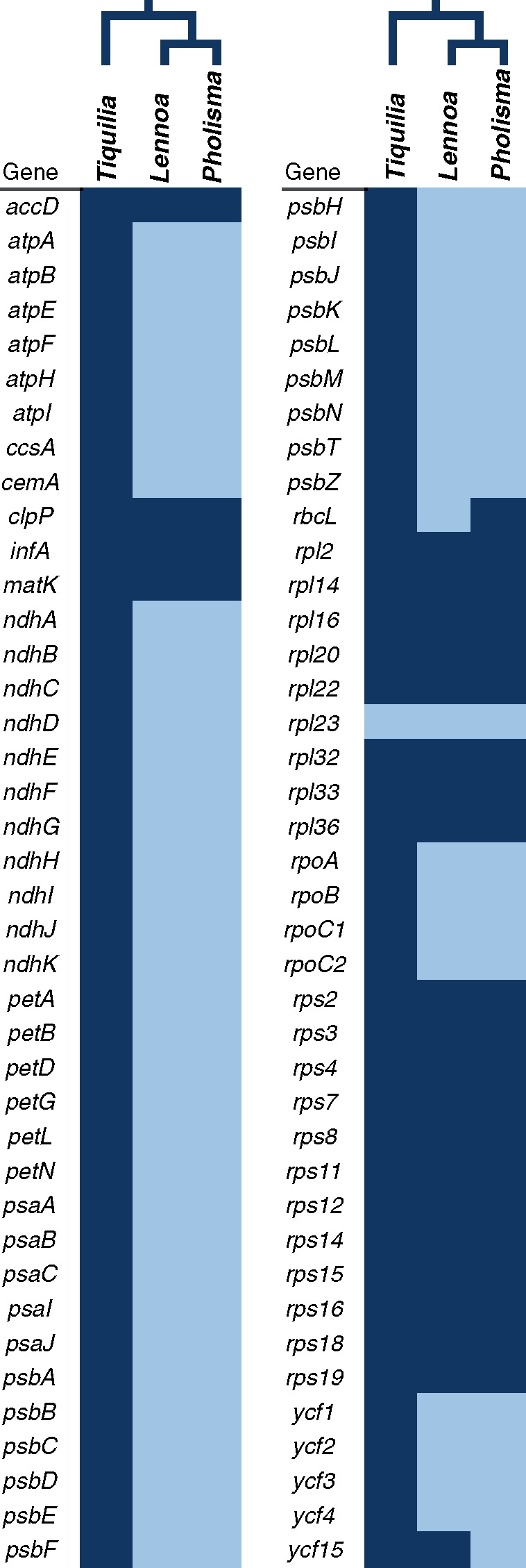
—Heat map showing plastome sequence content in *Tiquilia plicata*, *Lennoa madreporoides*, and *Pholisma arenarium*. Genes represented in dark blue are present and presumed fully functional; those in light blue are absent or pseudogenized. Phylogenetic relationships are indicated above the genus names.

The chloroplast genomes of *P.arenarium*, *L.madreporoides*, and the autotrophic relative *T.plicata* were assembled with high coverage as circular molecules and submitted to GenBank (table 2). The plastome of *T. plicata* is very similar in structure, gene content, and synteny to canonical plastomes of other autotrophic eudicots though two small frameshift mutations (likely duplications) have resulted in the pseudogenization of *rpl23* (table 2, fig. 1). Plastomes of both *P. arenarium* and *L. madreporoides* were nearly 50% smaller in size than that of *T. plicata*, and most of the sequence loss concentrated in the large and small single copy regions (LSC, SSC) (table 2, fig. 1). Similarly, of the 114 genes identified in *T. plicata*, 54 (47%) have been pseudogenized or lost in *P. arenarium* and *L. madreporoides* (fig. 2). Genes absent from both parasite species (but present in *T. plicata*) include all NADH dehydrogenase (*ndh*), *pet*, and photosystem I and II (*psa*, *psb*) genes*.* Although both parasitic species appear to have *psaI*-like ORFs, the high divergence at the amino acid level and reduced length relative to the putatively functional and highly conserved copy in *T. plicata* and other autotrophic angiosperms indicates that this gene is likely not functional in either parasitic species. In contrast, all plastid-encoded ribosomal protein (*rpl, rps*), rRNA, and tRNA genes are intact in all three species, with the exception of trnV^UAC^, which has been lost from both *P. arenarium* and *L. madreporoides*, and *rpl23*, which is pseudogenized in all three species, although the lack of clear synapomorphies suggest this may this be a result of convergence. In *L. mardreporoides*, the length of the pseudogenized *rbcL* has been reduced by nearly a third (1018 bp vs. 1476 bp in *P. arenarium*) due to several large and numerous small indels. Additionally, ORFs of *ycf15*, a gene of unknown function, are present in *T. plicata* and *L. madreporoides* but not *P. arenarium* due to a 5 bp insertion (duplication). Finally, although three species retain an ORF for *accD*, this gene is 4% shorter in *P. arenarium* and *L. madreporoides* due to several in-frame deletions.

**Table 2:** Plastid Genome Size and Structure of Lennoaceae Species and Autotrophic Relative *Tiquilia plicata*

	*Tiquilia plicata*	*Lennoa madreporioides*	*Pholisma arenarium*
Plastome size (bp)	154,559	83,675	81,198
Coverage	846×	52×	615×
GC%	37.5	37.1	38.1
Large single copy region (bp [%])	85,835	30,881	30,262
	(55.5)	(36.9)	(37.3)
Small single copy region (bp [%])	18,290	6,830	6,454
	(11.8)	(8.2)	(7.9)
Inverted repeat (bp [%])	25,217	22,982	22,241
	(16.3)	(27.5)	(27.4)
Gene content (protein coding/tRNA/rRNA)	114 (80/30/4)	60 (27/29/4)	60 (27/29/4)
GenBank accession	MG573056	MH237602	MH237601

### Plastome Structural Evolution

Gene order is identical in *T. plicata* and *L. madreporoides* within the LSC, SSC and the IR. However, in *P. arenarium* we found two rearrangements in a portion of the large single copy region rich in tRNA encoding genes relative to the other two species sampled (as well as most other angiosperms). First, the fragment 5′–trnE^UUC^—trnY^GUA^—trnD^GUC^—*ΨpetN*–3′ is inverted and translocated between trnS^UGA^ and trnG^GCC^. Second, 5′–trnL^UAA^—trnF^GAA^–3′ is inverted (fig. 1). The LSC/IR boundary is within *rps19* in *T. plicata* and *P. arenarium*, but the IR encompasses all of *rps19*, all of *rps22*, and part of *rps3* in *L. madreporoides* (fig. 1). The most parsimonious explanation is that this IR expansion followed the divergence of *P. arenarium* and *L. madreporoides.*

### Selection

**Fig. 3.**
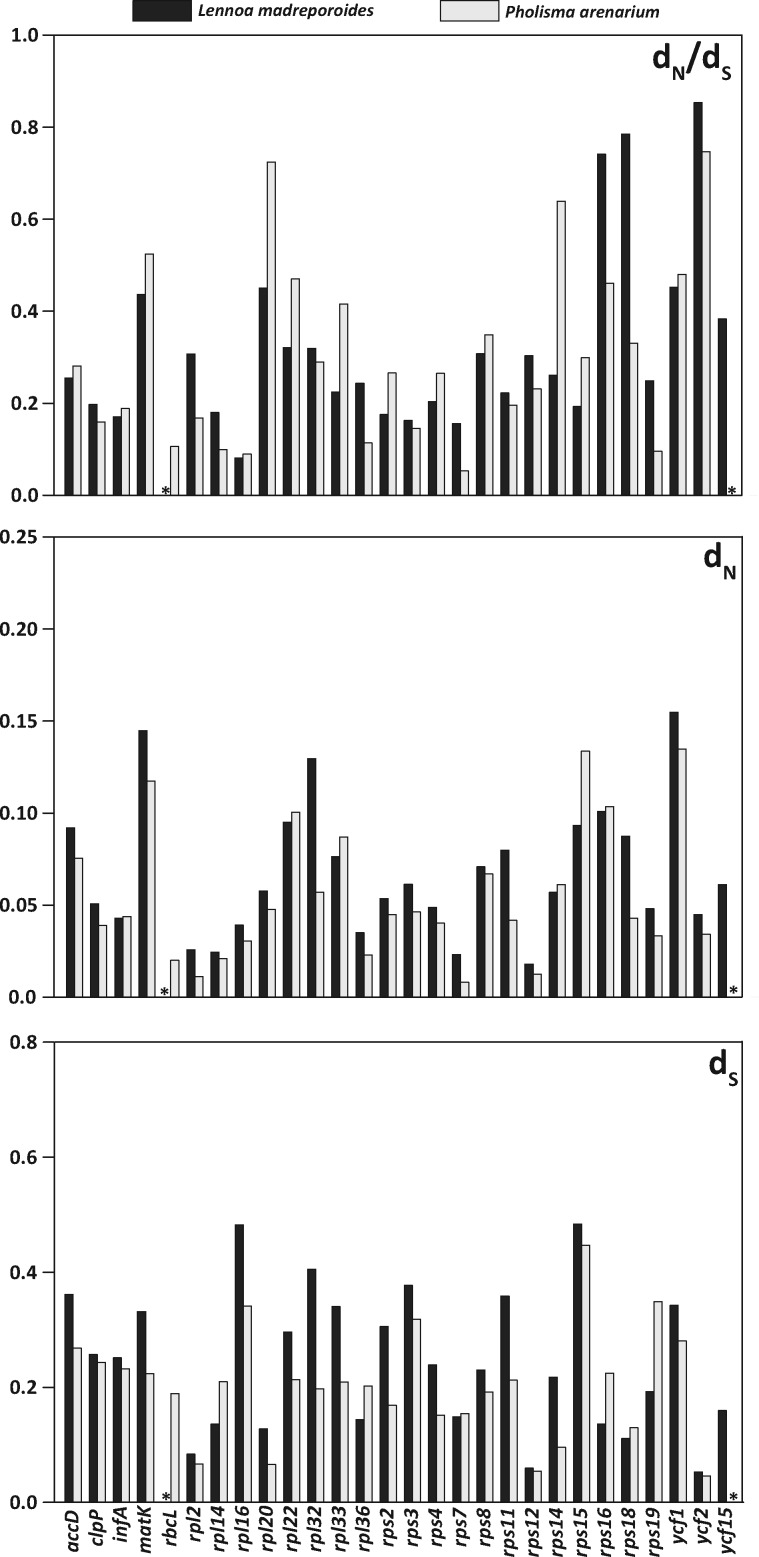
—Bar plot showing the ratio of nonsynonymous to synonymous substitutions (*d*_N_/*d*_S_) and the numbers of nonsynonymous (*d*_N_) and synonymous substitutions (*d*_S_) per site for Lennoaceae species relative to their autotrophic relative *Tiquilia plicata*. Dark grey bars represent values for *Lennoa madreporoides* and light grey for *Pholisma arenarium*. An asterisk indicates absence of a putatively functional gene copy.

High *d*_N_/*d*_S_ ratios (0.7 <  *d*_N_/*d*_S_ < 1) were observed for *rps16*, *rps18*, and *ycf2* in *L. madreporoides* but only *rpl20* and *ycf2* in *P. arenarium* (table 3, fig. 3). We do observe some relaxation of selection (0.3 <  *d*_N_/*d*_S_ < 0.7) for both *L. madreporoides* and *P. arenarium* in *matK*, *rpl22*, *rpl32*, *rps8*, and *ycf1*. Genes under purifying selection (*d*_N_/*d*_S_ < 0.3) for both *L. madreporoides* and *P. arenarium* include *accD*, *clpP*, *infA*, *rpl14*, *rpl16*, *rpl36*, *rps2*, *rps3*, *rps4*, *rps7*, *rps11, rps15*, and *rps19*. The gene *rbcL* in *P. arenarium* is under purifying selection (*d*_N_/*d*_S_ = 0.11). Although the *d*_N_/*d*_S_ ratio of *psaI* in *L. madreporoides* suggests it could be under strong purifying selection (*d*_N_/*d*_S_ = 0.26), high substitution rates suggest that this gene may not be functional at all or is evolving neutrally.

**Table 3:** The Ratio of Nonsynonymous to Synonymous Substitutions (*d*_N_/*d*_S_) and the Numbers of Nonsynonymous (*d*_N_) and Synonymous Substitutions (*d*_S_) per site for Lennoaceae Species Relative to Their Autotrophic Relative *Tiquilia plicata*

	*d*_N_/*d*_S_		*d*_N_		*d*_S_	
gene	*Lennoa madrepoioides*	*Pholisma arenarium*	*Lennoa madrepoioides*	*Pholisma arenarium*	*Lennoa madrepoioides*	*Pholisma arenarium*
*accD*	0.25	0.28	0.09	0.08	0.36	0.27
*clpP*	0.20	0.16	0.05	0.04	0.26	0.24
*infA*	0.17	0.19	0.04	0.04	0.25	0.23
*matK*	0.44	0.52	0.14	0.12	0.33	0.22
*rbcL*	0.26	NA	0.21	NA	0.78	NA
*rpl2*	NA	0.11	NA	0.02	NA	0.19
*rpl14*	0.31	0.17	0.03	0.01	0.08	0.07
*rpl16*	0.18	0.10	0.02	0.02	0.14	0.21
*rpl20*	0.08	0.09	0.04	0.03	0.48	0.34
*rpl22*	0.45	0.72	0.06	0.05	0.13	0.07
*rpl32*	0.32	0.47	0.10	0.10	0.30	0.21
*rpl33*	0.32	0.29	0.13	0.06	0.41	0.20
*rpl36*	0.22	0.42	0.08	0.09	0.34	0.21
*rps2*	0.24	0.11	0.04	0.02	0.14	0.20
*rps3*	0.18	0.27	0.05	0.04	0.31	0.17
*rps4*	0.16	0.15	0.06	0.05	0.38	0.32
*rps7*	0.20	0.26	0.05	0.04	0.24	0.15
*rps8*	0.16	0.05	0.02	0.01	0.15	0.15
*rps11*	0.31	0.35	0.07	0.07	0.23	0.19
*rps12*	0.22	0.20	0.08	0.04	0.36	0.21
*rps14*	0.30	0.23	0.02	0.01	0.06	0.05
*rps15*	0.26	0.64	0.06	0.06	0.22	0.10
*rps16*	0.19	0.30	0.09	0.13	0.48	0.45
*rps18*	0.74	0.46	0.10	0.10	0.14	0.22
*rps19*	0.79	0.33	0.09	0.04	0.11	0.13
*ycf1*	0.25	0.10	0.05	0.03	0.19	0.35
*ycf2*	0.45	0.48	0.15	0.13	0.34	0.28
*ycf15*	0.85	0.75	0.04	0.03	0.05	0.05

## Discussion

We present here fully sequenced and annotated plastomes of two of the four accepted species in the Lennoaceae—*L.madreporoides* and *P.arenarium*—along with the plastome of *T.plicata*, a closely related autotrophic species sequenced as a reference. Overall, the pattern of gene loss following the evolution of parasitism in the Lennoaceae is similar to that seen in other lineages. For example, complete or near-complete loss of *ndh, psb* and *psa* genes has been observed in holoparasitic Hydnoraceae, Cynomoriaceae, Apodanthaceae, Cytinaceae, and Orobanchaceae (Wicke et al. 2013; Bellot and Renner 2015; Bellot et al. 2016; Naumann et al. 2016; Roquet et al. 2016), and nonphotosynthetic mycoheterotrophic Orchidaceae and Ericaceae (Braukmann et al. 2017; Barrett et al. 2018). It is likely that these gene losses happened in a common ancestor of all Lennoaceae. This could be confirmed by sampling the two Lennoaceae species not investigated here (*P.sonorae* and *P. culiacanum*). However, given the similarity of plastomes between the more distantly related *L.madreporoides* and *P.arenarium* (hereafter referred at the generic level for ease of reading) we expect that this would not provide substantial additional insights.

The majority of genes remaining in the plastomes of *Lennoa* and *Pholisma* encode ribosomal proteins (*rpl* and *rps* genes, figs. 1 and 2). Most of these genes are under purifying selection as they are essential for translation of genes not involved in photosynthesis (e.g., *accD*, fig. 3, table 3). Several ribosomal proteins appear to be evolving under relaxed selection (e.g., *rps16* and *rps 18* in *Lennoa*). The loss of these genes is not restricted to heterotrophic plants, as they are also lost frequently among autotrophic lineages due to replacement by nuclear analogues (Jansen et al. 2007; Ueda et al. 2008; Graham et al. 2017). Nonetheless, the loss of *rpl* and *rps* genes appears to be accelerated in some heterotrophic plants (Naumann et al. 2016; Braukmann et al. 2017). Like other heterotrophic plants, the large genes of unknown function *ycf1* and *ycf2* are present in Lennoaceae, and putatively functional. However, only *ycf2*, likely an ATPase, is evolving under relaxed selection in both *Lennoa* and *Pholisma*, whereas we find *ycf1* under weak purifying selection. On the other hand, *d*_N_ and *d*_S_ rates are low for *ycf2*, suggesting a low rate of nucleotide substitution, typical for genes in the IR region.

The persistence of *rbcL* under purifying selection in *Pholimsa*, and the loss of this gene in *Lennoa*, parallels the evolutionary history of *Aphyllon* and *Harveya* in the Orobanchaceae. In these lineages, intact *rbcL* ORFs are retained (and at least transcribed in *Harveya*), but the gene is pseudogenized in their respective holoparasitic sister genera *Phelipanche* and *Hyobanche* (Leebens-Mack and dePamphilis 2002; Randle and Wolfe 2005). Similarly, *rbcL* is often lost in mycoheterotrophic lineages, but has been notably retained in *Pleuriscospora fimbriolata* (Braukmann and Stefanović 2012). Various hypotheses have been proposed to explain why this gene may be retained in putatively nonphotosynthetic plants, including involvement in amino acid synthesis via the gylcolate pathway, or regulating and recycling respirated CO_2_ (Bungard 2004; Randle and Wolfe 2005). The parallel maintenance of this gene in several independent lineages provides a minimal degree of evolutionary replication for future studies on *rbcL* gene expression and possible activity of RuBisCO in nonphotosynthetic plants.

Although *Lennoa* and *Pholisma* both appear to retain ORFs for the photosystem I gene *psaI* similar in length to the putatively functional copy in *T. plicata*, the high divergence at the amino acid level indicates that these are both likely pseudogenized. However, the fact that recognizable portions of the plastome still exist for this gene suggests that pseudogenization may have happened quite recently relatively to the other photosystem genes.

Current divergence time estimates based on plastid markers support a late Paleocene to early Eocene stem age for the Lennoaceae, and the most recent common ancestor of that clade with *Tiquilia* (Luebert et al. 2017). However, the crown age is much less certain, in part due to a lack of fossils and long molecular branch lengths within the Lennoaceae. Therefore, it is hard to precisely estimate the duration over which the photosynthesis-related genes were lost, or if genomic change since the divergence of *Lennoa* and *Pholisma* is proceeding as rapidly as the initial stages of plastome loss following parasitism. Evidence from other lineages of parasitic plants indicates that rate acceleration likely occurred prior to the loss of photosynthesis (Wicke et al. 2016; Barrett et al. 2018), though exactly when along the stem branch this occurred remains a mystery.

In conclusion, the objective of this study was to clarify the state of the plastome in the Lennoaceae, one of the last remaining unexplored independent lineages of parasitic angiosperms. Analysis of assembled plastomes from the holoparasites *L.madreporoides* and *P.arenarium* and comparisons with the closely related autotroph *T.plicata* demonstrate that parasites in the Lennoaceae exhibit convergent trends in sequence length reduction, relaxation of selection, and loss in gene content that have been observed in other heterotrophic plants. *Lennoa* and *Pholisma* have lost most plastid genes involved in coding for the photosynthetic apparatus while having retained the bulk of housekeeping genes and those that code for nonbioenergetic functions. This reinforces the idea of convergent molecular evolution between parasitic plants, not only within individual lineages of parasites, but also across the angiosperms.

## Note Added in Proof

After the acceptance of this manuscript, we became aware of an unpublished PhD dissertation by Yan Zhang (2012) [https://etda.libraries.psu.edu/catalog/16274] that, in part, covers some of the same topics as this paper. Zhang’s findings in Chapter 2 generally match those reported here.
